# Induction of apoptosis, oxidative stress, hormonal, and histological alterations in the reproductive system of thiamethoxam-exposed female rats

**DOI:** 10.1007/s11356-023-27743-2

**Published:** 2023-06-02

**Authors:** Marwa Alaa El-Din Sarry El-Din, Abd El-Wahab El Ghareeb, Islam M. El-Garawani, Heba Ali Abd El-Rahman

**Affiliations:** 1 Department of Zoology, Faculty of Science, Cairo University, Cairo, Egypt; 2 Zoology Department, Faculty of Science, Menoufia University, Shebin El-Kom 32511, Menoufia, Egypt

**Keywords:** Thiamethoxam, Reproductive hormones, Wistar rat, Apoptosis, Oxidative stress

## Abstract

The present study aimed to investigate the oral toxic effects of 1/10 LD_50_ and 1/5 LD_50_ of thiamethoxam (TMX), a neonicotinoid insecticide, on the reproductive system of female Wistar rats. Thirty female rats were divided into three groups and supplied orally with either; saline solution, 1/10 LD_50_ of TMX (156 mg/kg) or 1/5 LD_50_ of TMX (312 mg/kg). The daily administration was extended for 30 days. Investigating the parameters of oxidative stress, hormonal levels, histopathological alterations, and the apoptotic markers (P53, BAX, BCL-2, and caspase-3) was performed in the uterus and ovary of rats. Results showed significant changes in the body weight gain, and relative weight of the left and right ovaries and uterus. Moreover, luteinizing hormone (LH), estradiol (ED), and progesterone (PG) serum levels were not significantly altered following TMX oral administration. The level of follicle-stimulating hormone in the TMX-exposed group (156 mg/kg) was significantly increased; however, a significant decrease was observed in TMX-exposed animals (312 mg/kg). TMX induced significant oxidative stress in exposed groups by reducing the activities of superoxide dismutase (SOD), glutathione (GSH), and catalase (CAT), and elevating malondialdehyde (MDA) levels. Following hematoxylin and eosin staining, the microscopic examination revealed deteriorated luteal cells with vacuolation in the corpus luteum, a follicle containing a degenerated oocyte and degeneration/necrosis of the circular muscle layer with a high rate of apoptotic cells in TMX-exposed animals. TMX induced transcriptional alterations in apoptosis-related genes shifting towards the activation of the intrinsic apoptotic pathway. Collectively, results suggest the toxic effect of the TMX on the reproductive health of female Wistar rats.

## Introduction

Pesticides are one of the most dangerous agricultural agents, impacting mammalian and aquatic systems (de Oliveira et al. 2010; Feki et al. 2019; El-Garawani et al. 2021) causing some types of cancer, asthma, hormone disruption, hypersensitivity, hepatotoxicity, and other disorders (Van Maele-Fabry et al. 2010; Ben Amara et al. 2011; Feki et al. 2019). Since natural hormones are in charge of maintaining the homeostasis of processes like reproduction and development, pesticides can serve as endocrine-disrupting chemicals (EDCs) and interfere with their synthesis, secretion, transport, binding, action, or removal (Zoeller et al. 2012; Moreira et al. 2021).

Neonicotinoids are organic highly water-soluble insecticides consumed in over 120 countries (El-Garawani et al. 2022). However, despite the fact that neonicotinoids were once believed to have a short biological half-life, resent studies have revealed that these pesticides can persist in the environment for periods ranging from 1 day to almost 19 years (Katić et al. 2021; Costas-Ferreira and Faro 2021). In particular, neonicotinoids cause neurotoxicity in target insects by acting as strong agonists of nicotinic acetylcholine receptors (nAChRs) and their wide-scale usage nowadays leads to non-selective hazards for mammals and aquatic organisms (Doltade et al. 2019; Hathout et al. 2021). Thiamethoxam (TMX) is a second-generation neonicotinoid insecticide used on the majority of agricultural crops worldwide (Yang et al. 2021). TMX was one of the neonicotinoids most frequently found in fruits and vegetables (Yi et al. 2023), with detection rates of 51% and 53% in studies done in Hangzhou (China) and the US Congressional Cafeteria, respectively (Lu et al. 2018). According to a US Geological Survey investigation, THM was found in surface waters that were contaminated with neonicotinoids insecticides at a rate of 21% (Hladik and Kolpin 2016); TMX can cause fish to behave abnormally in response to environmental cues, which could have an impact on an ecosystem (Yang et al. 2023). TMX has been found in water sources all over the world, including drinking water (Chen et al. 2018a, b), subterranean (Davranche et al. 2019), and surface water (Chrétien et al. 2017; Casillas et al. 2022). A 16 eastern Chinese rivers have TMX. The Pearl River had 4.97–102 ng/L TMX. The USA has 8.93 g/L TMX remnants, Germany 2.82 g/L, and the Netherlands 225 g/L (Yang et al. 2023). Additionally, the low reference dose of TMX points to a high relative toxicity of TMX (Jameel et al. 2020). Thus, it poses an issue when considering the potential for occupational and environmental contamination.

In rats, TMX metabolism is converted to nitrosoguanidine, aminoguanidine, guanidine, and urea derivatives (Hataba et al. 2014) and has the potential to cause severe disruptions in the liver functions. Moreover, TMX exposure was associated with pronounced deleterious effects on renal, hepatic, cardiac, and testicular functions (El Okle and Lebda 2016). Hepatotoxicity associated with the appearance of necrotic foci and oxidative stress was reported by sub chronic doses of TMX in male rats (Auwal et al. 2021). Furthermore, the kidney, liver, and brain histopathological damage as well as the systemic biochemical alterations were observed in sub chronic oral administration of TMX in male rats (Khaldoun-Oularbi et al. 2017). In male rats, fertility may be adversely affected by TMX exposure through alteration of spermatogenesis, steroidogenesis, testicular redox status, and testicular DNA damage (Abd-Allah et al. 2022). The adverse effects in testicular function and structure of TMX-exposed rabbits were reported too (ElSawasany et al. 2017). Although the data about the action of TMX on nAChRs receptors in insects is clear, to our knowledge, there was no clear data about the possible effect of TMX exposure on the reproductive system of female Wistar rats. Thus, the present study was carried out to evaluate the hazardous effects of TMX oral administration on the reproductive system of female Wistar rats focusing on the histological, hormonal, and molecular parameters.

## Materials and methods

### Chemicals

Thiamethoxam, CAS No. 153719–23-4 (≥ 98% purity), has a formal name of 3-(2-chloro-thiazol-5-ylmethyl)-5-methyl-[1,3,5]oxadiazinan-4-ylidene-N-nitroamine) and C_8_H_10_ClN_5_O_3_S molecular formula (Cayman Chemicals, MI, USA).

### Animals

Female Wistar rats, *Rattus norvegicsus*, weighing 165±5 gm was purchased from the animal house of the Faculty of Veterinary, Cairo University, Egypt. Rats were kept under the laboratory conditions of 20–25 °C and 12-h dark/light cycle for 1 week as an acclimatization period. They were housed in special healthy standard cages.

### Experimental design

Eighteen female rats were randomly divided into three groups (6 rats/ group). Group (1) animals were served as a negative control group and given saline solution as a vehicle. Group (2) animals received 1/10 LD_50_ of TMX (156 mg/kg). The third group received 1/5LD_50_ TMX (312 mg/kg). All groups were administered the treatments by oral gavage daily. On day 31, rats were sacrificed under isoflurane anesthesia.

### General health

The mortality, body weight, and clinical signs of toxicity were recorded during the period of the experiment.

Rat’s body weight was examined after TMX intake ovary and uterus were dissected, examined, and weighed to calculate the absolute and relative weight according to the following formula:

$$\begin{array}{c}\mathrm{Body\ weight\ change }\left(\mathrm{g}\right)=\left[\mathrm{final\ body\ weight\ }(\mathrm{g})-\mathrm{initial\ body\ weight\ }(\mathrm{g})\right]\\ \mathrm{Relative\ organ\ weights }\left(\mathrm{\%}\right)=\left[\mathrm{organ\ weight }(\mathrm{g})/\mathrm{body\ weight\ }(\mathrm{g})\right]\times 100\end{array}$$

### Hormonal analysis

Blood was collected in a serum-collection tube without anticoagulant and allowed to coagulate at room temperature for about 20 min before being centrifuged at 3000 rpm for 15 min. The supernatant serum samples were then transferred to dry clean-capped tubes and stored at − 20 °C until the hormonal analysis was performed, namely, serum follicle stimulating hormone (FSH), luteinizing hormone (LH), estradiol (ED), and progesterone (PG) according to the instructions of Fine Test ELISA kit specific for each hormone Wuhan (Fine Biotech Co., Wuhan, Hubei, China).

### Oxidative stress investigation

To assess the oxidative stress caused by TMX, ovary and uterus tissue samples were collected from control and treated rats, washed in 0.05 M phosphate buffer (pH 7), then dried by blotting between the folds of a filter paper. The tissue homogenate (10%, wt/v) was prepared in 0.05 M phosphate buffer (pH 7) using a polytron homogenizer (KINEMATICA -POLYTRON^®^PT 1300 D- PB (230 V), Switzerland) at 4 °C. The homogenate was centrifuged at 10,000 rpm for 20 min for removing the cell debris, unbroken cells, nuclei, erythrocytes, and mitochondria. The supernatant (cytoplasmic extract) was used for the estimation of superoxide dismutase (SOD), glutathione (GSH), catalase (CAT), and malondialdehyde (MDA) colorimetrically according to manual instructions (BioVision’s Assay Kit, USA). Protein content in the tissue was determined according to the method of Bradford (1976) using Genei, Bangalore, protein estimation kit. All ELISA kits were measured by ELISA reader Color absorbance was read at OD range 490 to 630 nm using an enzyme-linked immuno-sorbent assay (ELISA) plate reader (Stat Fax 2200, Awareness Technologies, FL, USA).

### Histopathological studies

Ovary and uterus samples were fixed in a 10% neutral formal saline solution for 24 h. After washing with tap water, dehydration was accomplished with repeated dilutions of 100% ethyl alcohol, cleared in xylol and paraffin wax tissue blocks were prepared for microtome sectioning at a thickness of 4 μm. The tissue sections were mounted on glass slides, deparaffinized, and stained with hematoxylin and eosin (H&E) for histological inspection using a light microscope (Olympus BX 41, Japan).

### Immunohistochemical blotting

For immunohistochemical evaluation of caspase-3 protein, 4-μm thickness of ovarian and uterine tissue sections on positive-charged slides was processed using the avidin–biotin peroxidase method (Hsu et al. 1981). Sections were incubated with a caspase-3 primary antibody for 60 min, a biotinylated secondary antibody, then with the standard horseradish peroxidase (HRP) conjugated with streptavidin for 15 min. Then, the 3-amino-9-ethylcarbasole (Dako Cytomation, USA) was added. Counterstaining with Mayer’s hematoxylin was done. The slides were examined, and the generated positive brown immuno-reaction was evaluated under an Olympus light microscope (BX 41, Japan) at 400 × magnification. The image analysis was done using a computer software, ImageJ (NIH, Bethesda, MD, USA) by evaluating the mean percentage area (%) of positive immunohistochemical reactions (3 fields/animal, *n* = 5).

### Quantitative real-time PCR

To measure Bcl-2, Bax, and P53 mRNA transcripts, the isolation of total RNA was done from tissues using RNeasy Plus Minikit (Qiagen, Hilden, Germany). The synthesized cDNA was obtained using the RevertAid™ H Minus Reverse Transcriptase (Thermo Fisher Scientific, Austin, US). Real-time PCR using Power SYBR® Green (Life Technologies, Carlsbad, CA, USA) was performed on an Applied Biosystems 7500 system (Foster City, CA, USA). GAPDH expression was used to normalize the relative values of gene expression. The used primer sequences and accession numbers of genes are shown in Table 1.

**Table 1:** The sequences of used primers

Name	Accession number	Sense (5′–-3′)	Antisense (5′–-3′)
GAPDH	NM_017008	TGCACCACCAACTGCTTAGC	GGCATGGACTGTGGTCATGAG
Bax	NM_017059.2	CCAGGACGCATCCACCAAGAAGC	TGCCACACGGAAGAAGACCTCTCG
Bcl-2	NM_016993.1	TATATGGCCCCAGCATGCGA	GCTGAGCAGCGTCTTCAGAGA
P53	AH002222.2	GTC GGC TCC GAC TAT ACC ACT ATC	CTC TCT TTG CAC TCC CTG GGG G

*GAPDH*, glyceraldehyde-3-phosphate dehydrogenase; *Bcl-2*, B-cell lymphoma 2; *Bax*, Bcl-2-associated X protein

### Statistical analysis

The mean of replicates was calculated, along with the standard deviation (SD) for each group. ANOVA with the Tukey test was used to make statistical comparisons (ANOVA). SPSS (SPSS, 1990) computer program was used to calculate the significance between groups in the same experiment (*P* < 0.05).

## Results

### General health and total body, ovarian and uterine weights

The TMX-treated female rats (156 and 312 mg/kg/b.wt) revealed no external symptoms of toxicity and mortality during the experiment. Furthermore, there was a significant decrease in the body weight gain between treated animals compared to untreated controls. However, there was a non-significant increase in the relative weight of ovaries and uterus in TMX-exposed animals respecting the untreated controls (Table 2).

**Table 2:** Body weight gain (g), relative ovarian, and uterine weights of female rats after oral administration of TMX for 30 days

Groups	Control	TMX-1/10 LD_50_	TMX-1/5 LD_50_
Body weight gain (g)	247 ± 9.24	4.50 ± 14.67^a^	1.5 ± 6.38^a^
Relative weight right ovary	0.028 ± 0.004	0.04 ± 0.004	0.035 ± 0.010
Relative weight left ovary	0.03 ± 0.01	0.038 ± 0.008	0.037 ± 0.008
Relative weight uterus	0.215 ± 0.06	0.298 ± 0.084	0.23 ± 0.079

Values are expressed as mean ± SD (*n* = 6). a indicates significance (*P* < 0.05). TMX-1/10 LD_50_, 156 mg/kg; TMX-1/5 LD_50_, 312 mg/kg

### Effect of TMX on female reproductive hormones

The assessment of serum hormonal parameters was performed, and results revealed that luteinizing hormone (LH) and progesterone (PG) were significantly decreased (*P*<0.05) in TMX-administrated female rats at doses of 156 and 312 mg/kg/b.wt by ~ 35 and 45% for LH and ~ 70 & 58% for PG respectively, compared to controls. Furthermore, compared to untreated groups, there was about 30% increase (*P*<0.05) in serum follicle stimulating hormone (FSH) in animals exposed to 156 mg/kg/b.wt dose of TMX; however, a significant decrease (~23%) was noticed in animals exposed to higher dose of TMX (312 mg/kg/b.wt). Furthermore, a non-significant increase (*P*>0.05) in serum estradiol (ED) was observed in animals exposed to both doses of TMX (156 and 312 mg/kg/b.wt) compared to controls (Table 3).

**Table 3:** Changes in serum follicle stimulating hormone (FSH), luteinizing hormone (LH), estradiol (ED), and progesterone (PG) in TMX-administered female rats for 30 days

Groups	Control	TMX-1/10 LD_50_	TMX-1/5 LD_50_
FSH (mIu/mL)	1 ± 0.294	1.3 ± 0.0.294^a^	0.775 ± 0.095^a^
LH (mIu/mL)	19.75 ± 2.34	13.32 ± 2.99^a^	11.1 ± 0.89^a^
ED (pg./mL)	20.07 ± 10.31	21.2 ± 16	21.3 ± 11.98
PG (ng/mL)	16.62 ± 4.18	4.94 ± 2.68^a^	6.94 ± 1.67^a^

Values are expressed as mean ± SD (*n* = 6). a indicates significance (*P* < 0.05). TMX-1/10 LD_50_, 156 mg/kg; TMX-1/5 LD_50_, 312 mg/kg

### Effect of TMX on ovarian tissue oxidative status

**Fig. 1:**
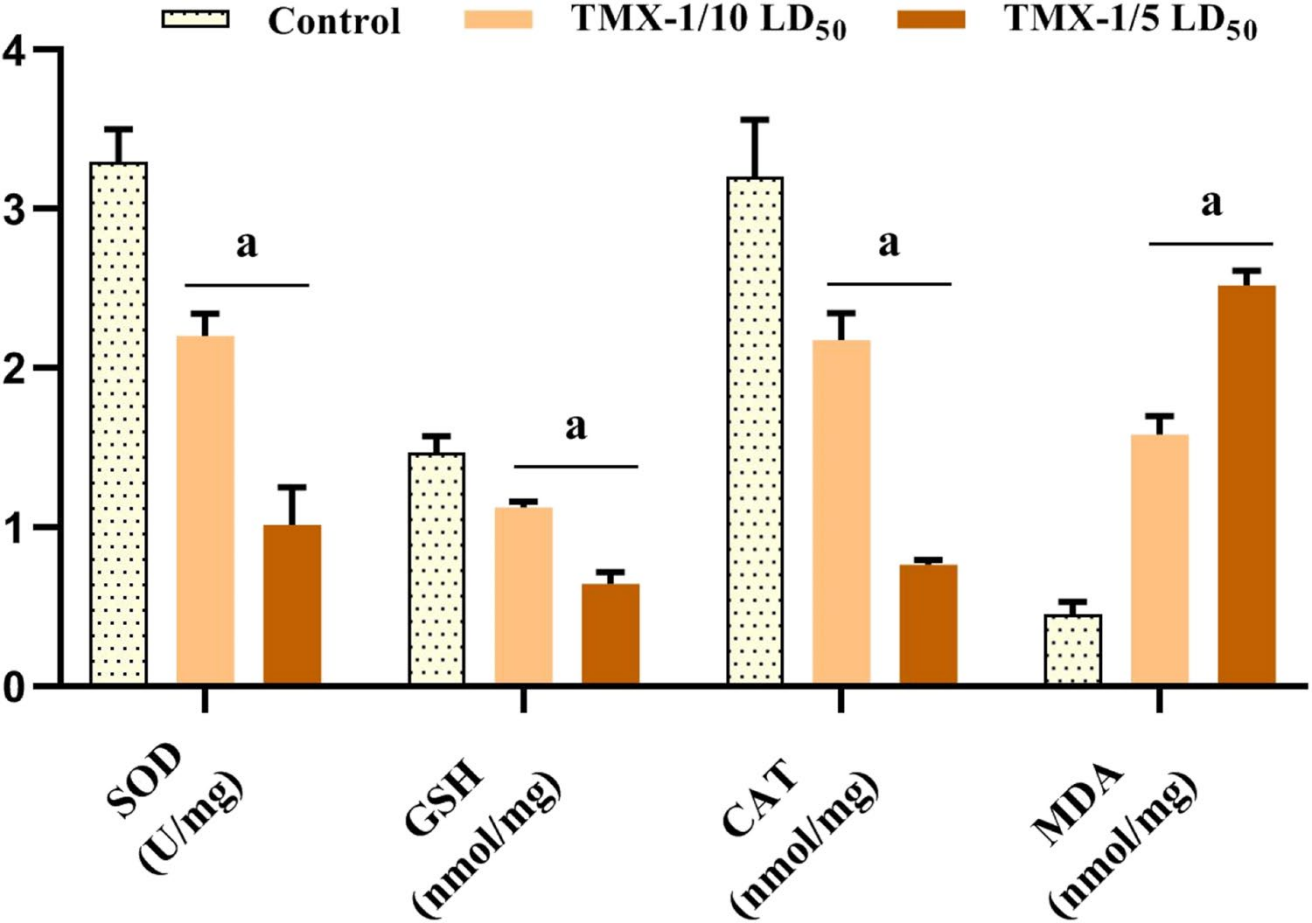
The effect of TMX on oxidative status of female rats’ ovary following the 30 days exposure. Values are expressed as mean ± SD (*n* = 6). a indicates significance (*P* < 0.05). TMX-1/10 LD_50_, 156 mg/kg; TMX-1/5 LD_50_, 312 mg/kg

Ovarian tissue oxidative status was evaluated in control and treated rats by measuring SOD, GSH, CAT, and MDA (Fig. 1). Compared to the controls, there was a significant dose-dependent reduction (*P* < 0.05) in the SOD, GSH, and CAT activities in animals treated with TMX by ~ 33.3, 24.3, and 32.2%, respectively, for the lower dose and ~ 69, 56.5, and 76%, respectively, for the higher one. However, when compared to untreated rats, a significant elevation was observed in MDA levels in both treated groups (~ 3.2 folds for the lower dose and ~ 5 folds for the higher one).

### Effect of TMX on uterus tissue oxidative status

**Fig. 2:**
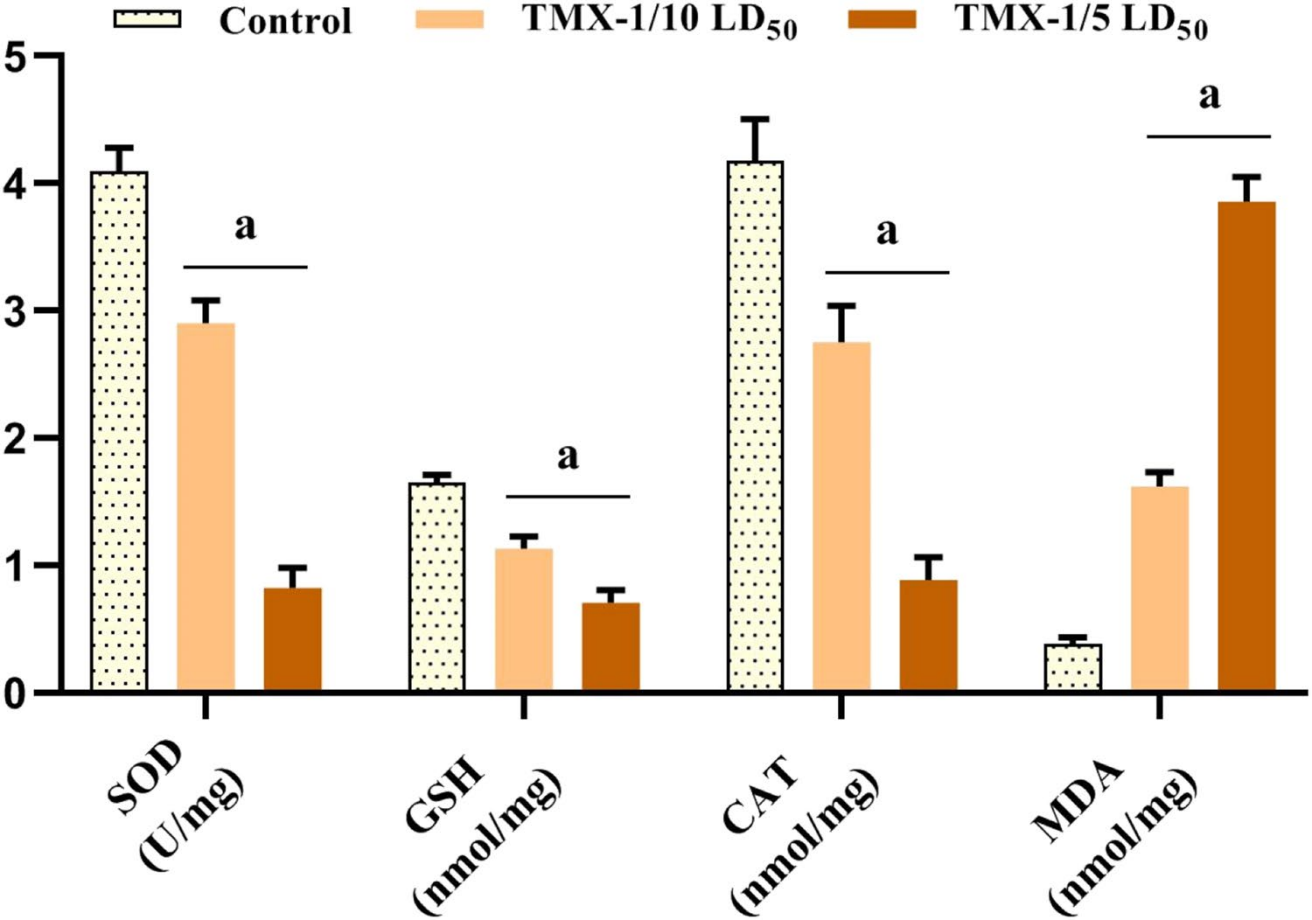
The effect of TMX on uterus tissue oxidative state of female rats for 30 days. Values are expressed as mean ± SD (*n* = 6). a indicates significance (*P* < 0.05). TMX-1/10 LD_50_, 156 mg/kg; TMX-1/5 LD_50_, 312 mg/kg

The oxidative status (SOD, GSH, CAT, and MDA) was investigated in the uterus of TMX-treated rats and controls (Fig. 2). Results revealed a significant dose-dependent reduction (*P* < 0.05) in the activities of SOD, GSH, and CAT in TMX-treated animals by ~ 29.2, 31.5, and 34%, respectively, for the lower dose and ~ 80, 57, and 78.7%, respectively, for the higher one compared to controls. However, a significant increase in MDA levels was noticed in both TMX-treated groups (~ 4.2 folds for the lower dose and ~ 10 folds for the higher one) when compared to the controls.

### Effect of TMX on ovarian histopathological outcomes

**Fig. 3:**
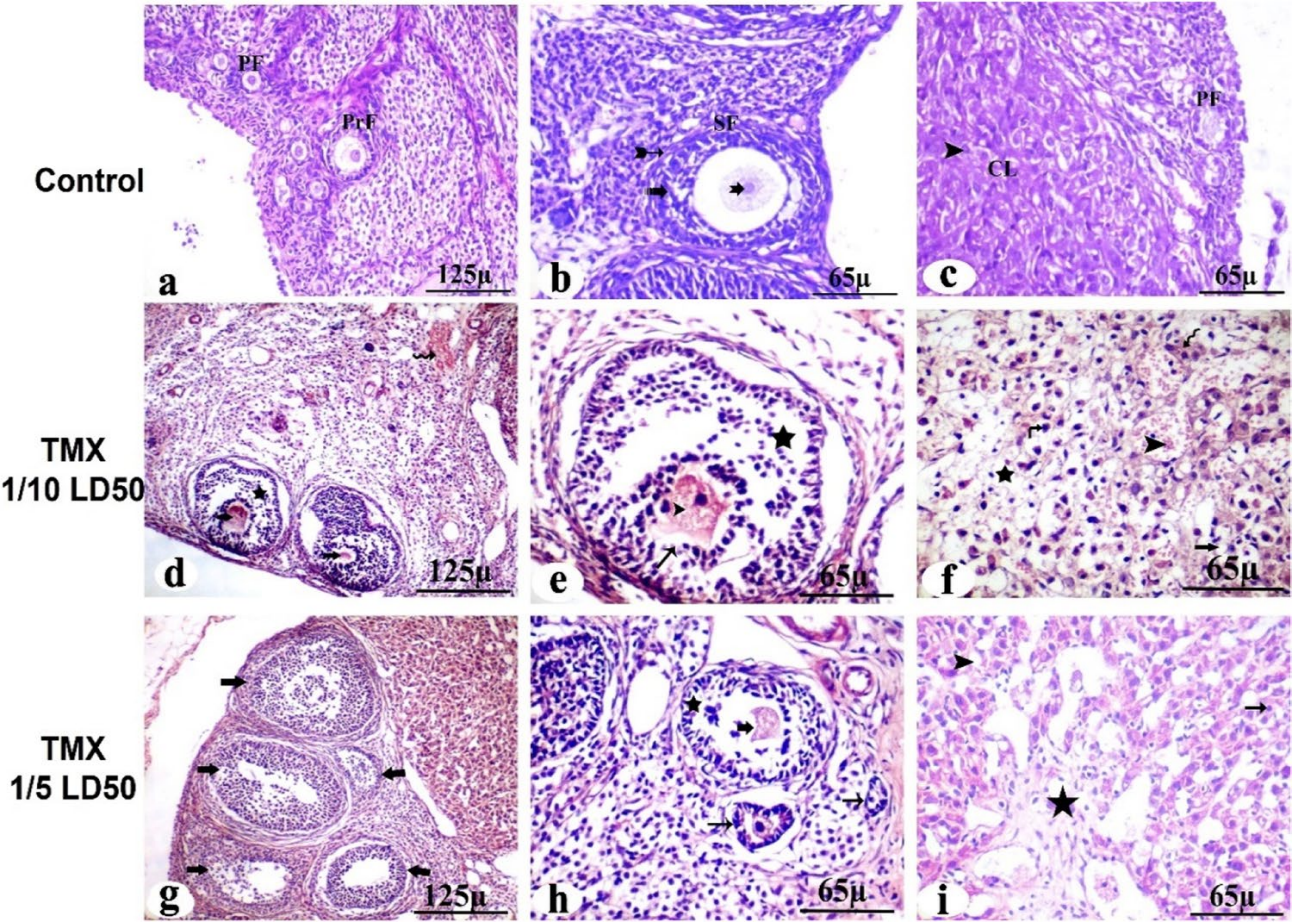
Photomicrographs of TMX-treated and control rats’ ovary Sects. (4 µm, H&E-stained). The presence of primordial follicular cells (PF), primary follicle (PrF), and mature secondary follicles (SF) that surrounded with theca follicle (bifid arrow) and contains viable oocytes (notch arrow) and granulosa layer with their nuclei (dotted arrow) (**a** and **b**, control). Large corpora lutea (CL) showing normal luteal cell (arrowhead) (**c**, control). Degeneration of pre-ovulatory follicle cells without oocytes (thick arrow-arrowhead) and disorganized granulosa layer (star) with deep acidophilic cytoplasm and pyknotic nuclei (thin arrow), massive hemorrhage in the ovarian stroma (wavy arrow) (**d** and **e**, low dose). Corpus luteum showing degenerative luteal cells (star) with vacuolation (arrows), congested area, pyknotic cell (curved arrow), and hyalinization (wavy arrow) (**f**, low dose). Abnormal secondary follicles without oocyte were observed (thick arrow) (**g**, high). Number of atretic follicles with degenerated follicular cells (thin arrow) and oocyte disinteegration (thick arrow) (**h**, high dose). Corpus luteum showing deteriorating luteal cells with vacuolation (arrowhead), necrotic area (star), pyknotic cell (thin arrow) (**i**, high)

The microscopic examination of control rats’ ovary sections (H&E-stained) showed the cortex containing the developing follicles. The growing follicles are characterized by their surrounding of granulosa cells. By examining the mature follicles, oocytes with round nuclei were surrounded by flattened follicular cells, ensheathed with multilayered granulosa cells and antral cavity. Atretic follicle and clusters of stromal cells were seen as well as large corpora lutea composed of moderately eosinophilic cells with large vesicular nuclei and foamy cytoplasm (Fig. 3a, b, c). Meanwhile, ovary of TMX-administered rats (156 mg/kg/b.wt) showed follicles consisting of degenerated oocytes and disorganized follicular granulosa cells with darkly stained nuclei, vacuolated stroma and hypercellularity stroma. Blood vessel walls were thickened, and smooth muscle cells were hypertrophied (Fig. 3d). Moreover, the observable reduction in ovarian follicles, degeneration of pre-ovulatory follicle cells without oocytes, and a disorganized granulosa layer degenerated oocyte (Fig. 3e). The corpus luteum showed degenerative cells with vacuolation, a congested area, pyknotic cells, and hyalinization (Fig. 3f). On the other hand, the ovary of TMX-administered rats (312 mg/kg/b.wt) showed a loss of normal ovarian histoarchitecture with multiple distorted and degenerated follicles, dark pyknotic nuclei, and congested dilated blood vessels. None of the follicles could be seen with intact ova and their normal nuclei (Fig. 3g). The atretic follicles, apoptotic cells with deep acidophilic cytoplasm, and pyknotic nuclei were seen. Some of the degenerated oocytes were encircled by a disrupted zona pellucida. In the ooplasm, vacuolation was observed (Fig. 3h). The corpus luteum contains deteriorating luteal cells with vacuolation, necrotic areas, and pyknotic cells (Fig. 3i).

### Effect of TMX on caspase-3 protein expression in ovarian tissues

**Fig. 4:**
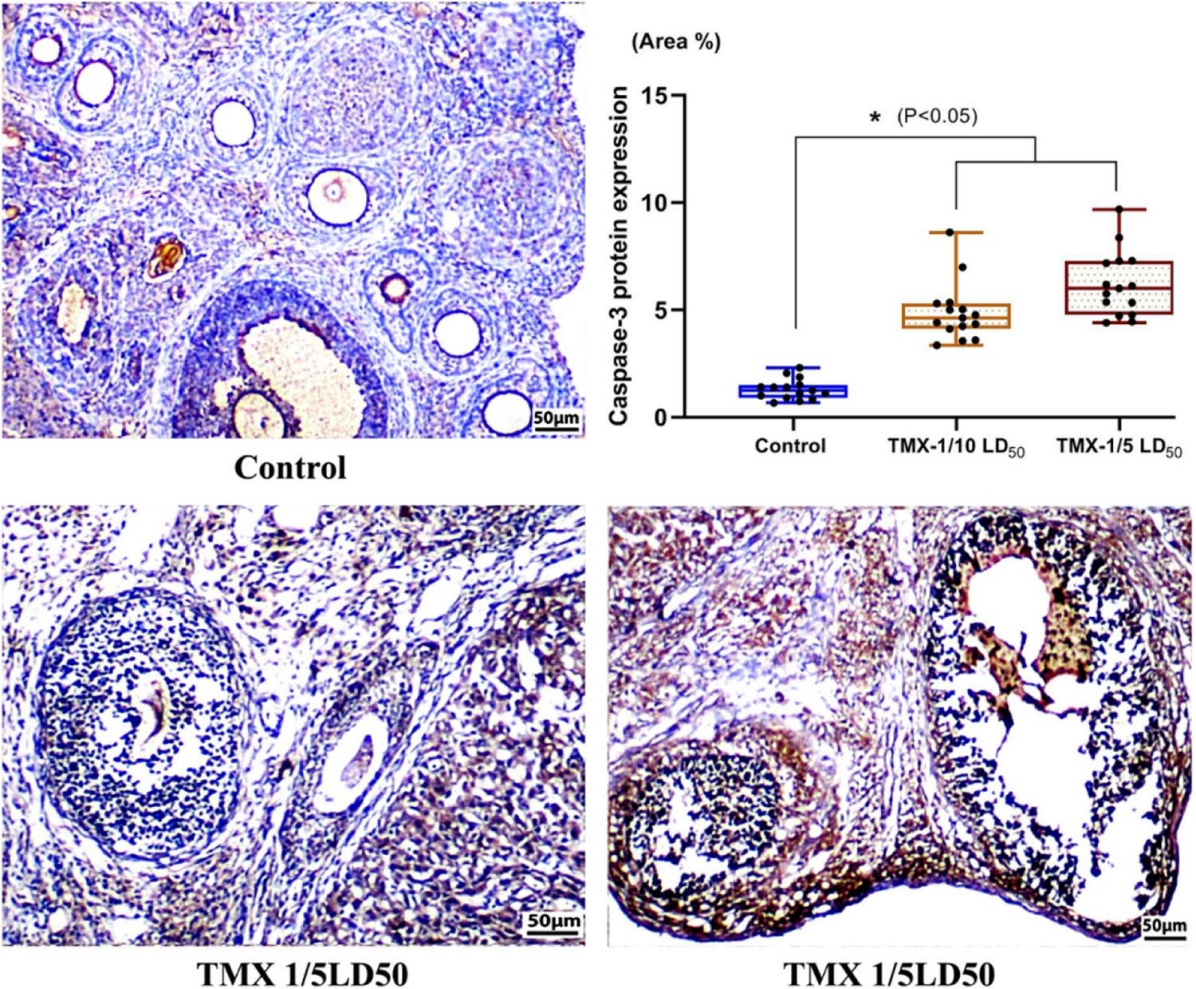
Photomicrographs of TMX-treated and control rats’ ovary Sects. (4 µm, caspase-3 immunoreactivity). The upper right pannel is a flouting bars illustrating the analysis of the reactive area percent (3 fields/animal, *n* = 5) using ImageJ software (1.46, USA)

The immunohistochemical reactivity of caspase-3 protein was evaluated in control and treated rats’ ovarian sections (Fig. 4). Results showed that TMX treatments caused a significant (*P* < 0.05) dose-dependent elevation in caspase-3 levels compared to controls. Relative to untreated rats, the levels of lower dose were increased by about 5.9 folds; however, the higher one showed about 9.8 folds.

### Effect of TMX on uterine histopathological outcomes

**Fig. 5:**
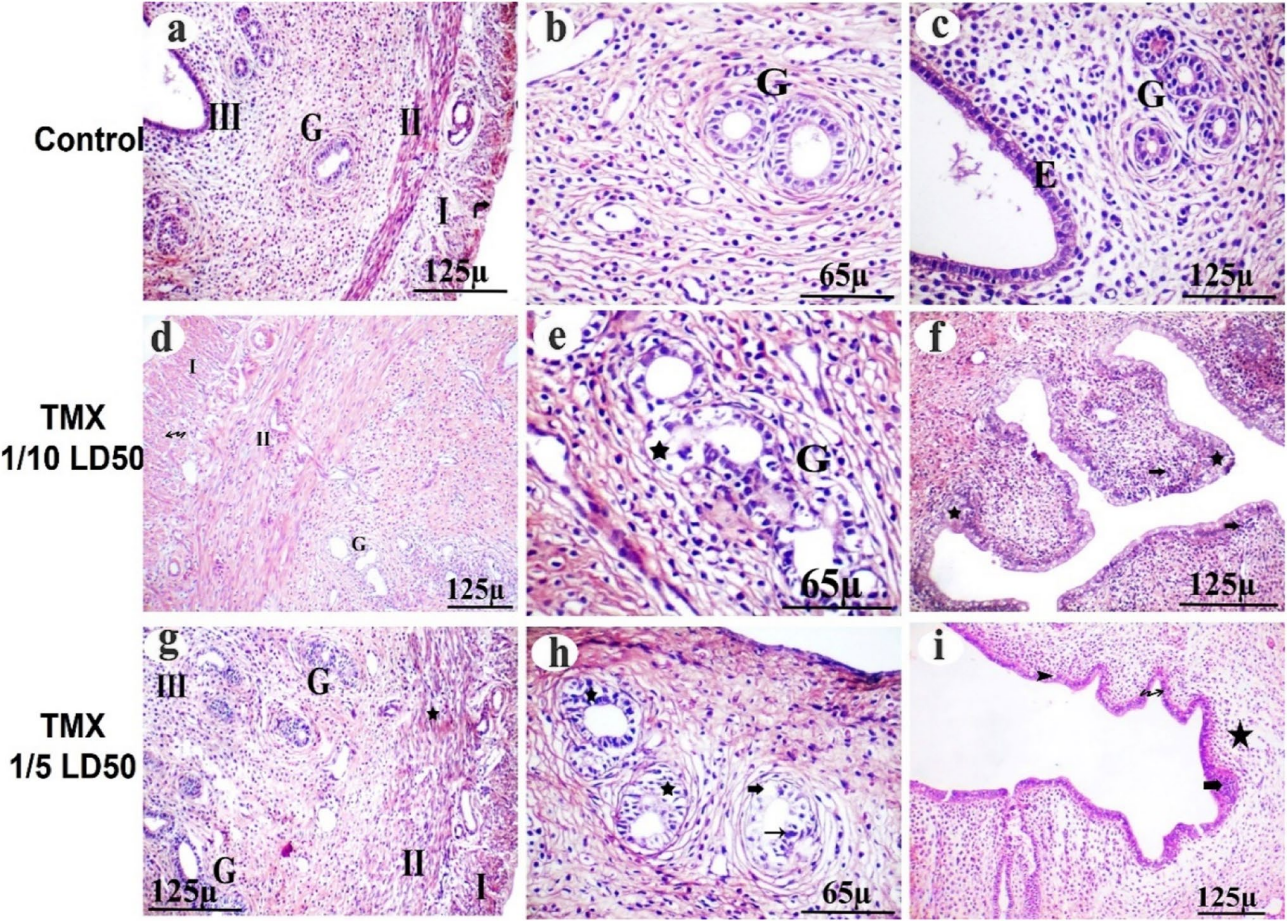
Photomicrographs of TMX-treated and control rats’ uterus Sects. (4 µm, H&E-stained) showing endometrium (III-E), longitudinal muscle (I), circular (II) and glandular epithelium (G) (**a**–**c**, control). Degeneration of the circular muscle layer with shrinkage-stained nuclei (II-star) and a high rate of apoptotic cells with multiple vacuolations (wavy arrow) (**d**, low dose). Branched glandular epithelium (G) with degenerated epithelial cells (star) (**e**, low dose), and severe neutrophils infiltration (arrow) in the endometrium and degenerated epithelium (star) were observed (**f**, low dose). The longitudinal muscle layer (I) showed signs of degeneration and vacuolation and disrupted (star) circular muscle (II). Endometrial glands (G) appeared to be larger than those in the control group (**g**, high dose). Degenerated glandular epithelial cells (star), vacuoles (bold arrow), and pyknotic nuclei (thin arrow) (**h**, high dose). The endometrium layer showed cytoplasmic vacuoles (arrowhead) and pyknotic nuclei (wavy arrow), degeneration (star), and lymphatic infiltration (thick arrow) (**i**, high dose)

Microscopically (H&E-stained), the uterus of control rats showed wall layers including endometrium, myometrium, longitudinal muscle, circular muscle, and perimetrium. Glandular epithelium can be observed (Fig. 5a–c). The presence of glands in the endometrium and glands lined with simple columnar cells with basal oval nuclei was seen (Fig. 5c). The uterus of TMX-administrated rats (156 mg/kg/b.wt) showed degeneration/necrosis of the circular muscle layer with a high rate of apoptotic cells, as well as a longitudinal muscle layer with deteriorated cells and multiple vacuolation (Fig. 5d). The uterine endometrium epithelium had degenerated cells and severe neutrophil infiltration in the endometrium and degenerated endometrium glands (Fig. 5e, f). However, the uterus of TMX-administrated rats (312 mg/kg/b.wt) showed signs of degeneration and vacuolation in the longitudinal muscle layer and circular muscle necrosis infiltrated by lymphocytes (Fig. 5g). Moreover, endometrial glands appeared to be larger, more branched, and have a wider lumen than those of untreated animals Apoptosis was also observed in the surface and glandular epithelial cells and appeared with deep acidophilic cytoplasm and pyknotic nuclei. Degeneration and vacuolation of glandular epithelial cells were observed too and degenerated endometrium epithelium with disrupted lamina propria (Fig. 5h, i).

### Effect of TMX on caspase-3 protein expression in uterine tissues

**Fig. 6:**
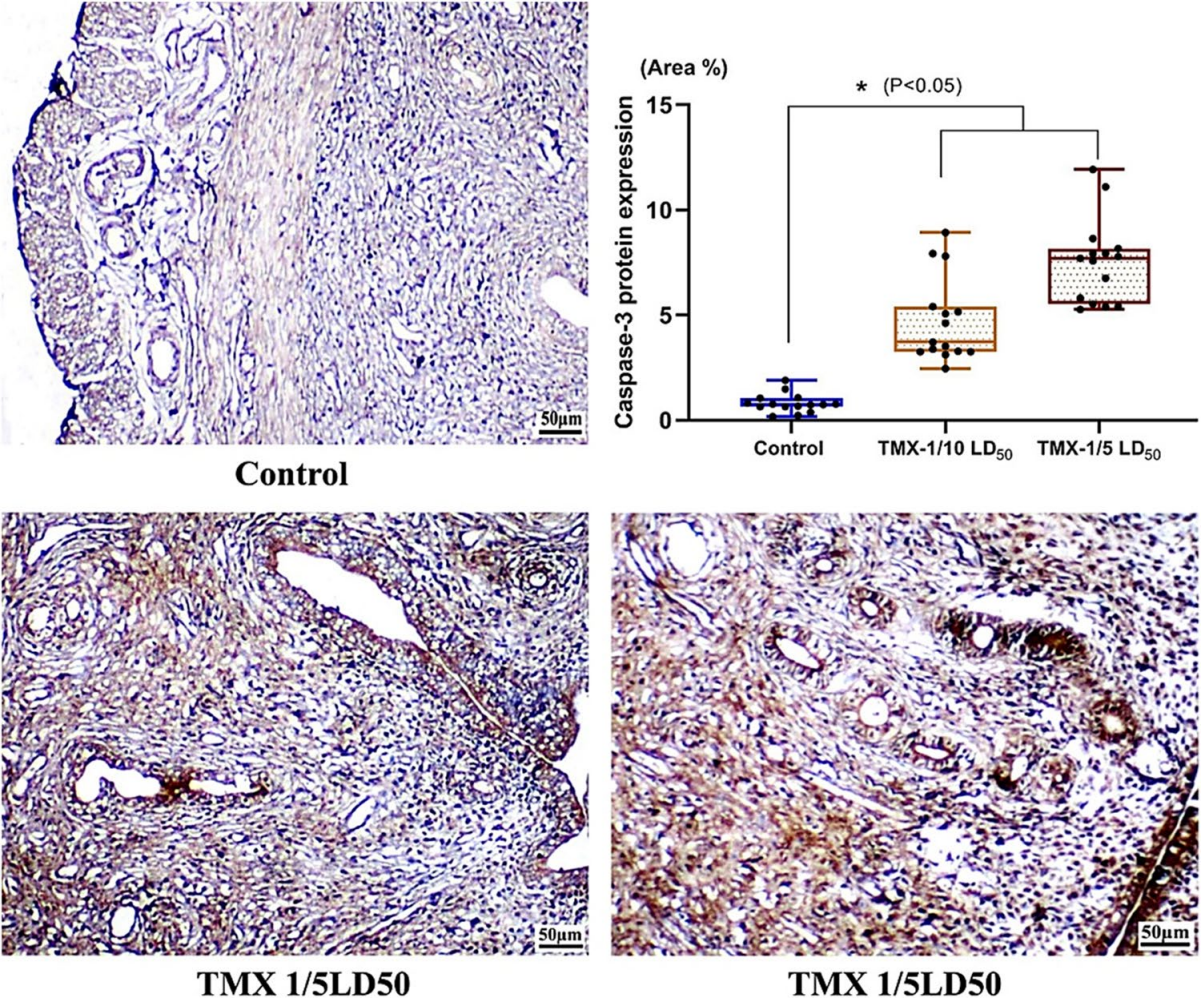
Photomicrographs of TMX-treated and control rats’ uterus Sects. (4 µm, caspase-3 immunoreactivity). The upper right panel is a flouting bars illustrating the analysis of the reactive area percent (3 fields/animal, *n* = 5) using ImageJ software (1.46, USA)

The immunohistochemical reactivity of caspase-3 protein was evaluated in control and treated rats’ uterine sections (Fig. 6). A significant (*P* < 0.05) dose-dependent elevation in caspase-3 levels compared to controls was observed in TMX-treated groups. Relative to untreated rats, the levels of lower dose were elevated by about 3.7 folds, and the higher one showed 4.8 folds.

### Altered transcription of apoptosis-related markers in TMX-treated ovary

**Fig. 7:**
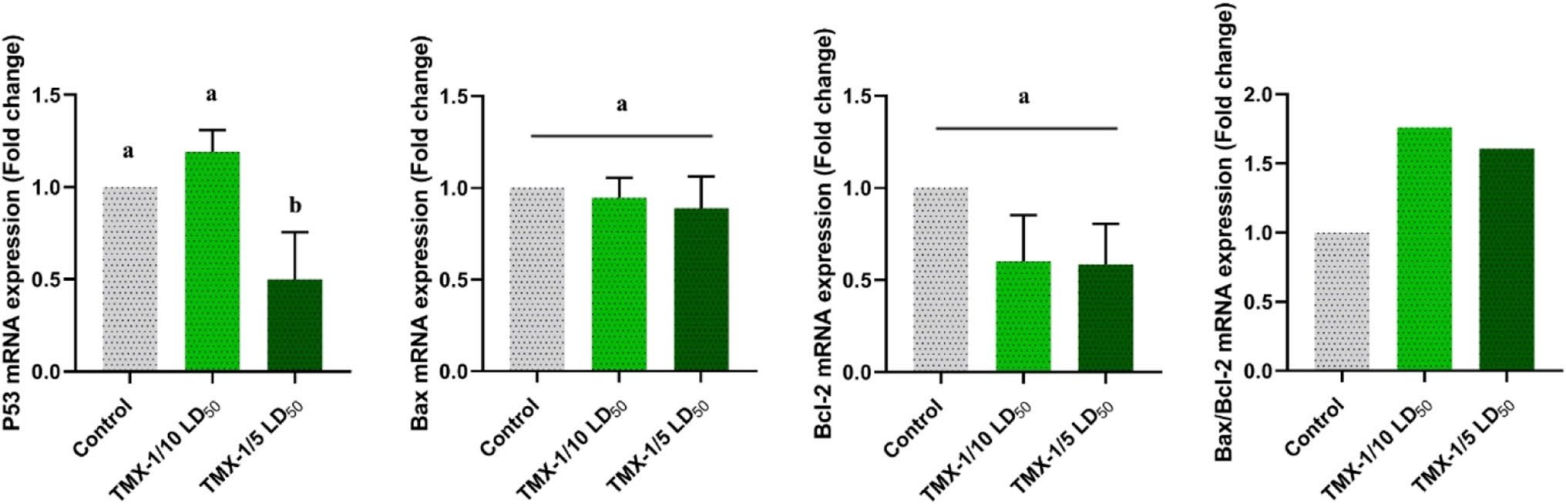
The effect of TMX on the relative gene transcription of Bax, Bcl-2, P53, and the ratio of Bax/Bcl-2 means in ovary tissues. Results were normalized to the GAPDH gene and calculated as a relative fold-change to the untreated group. Data were represented as mean ± SD (*n* = 5) and significance was represented by different letters (*P*˂0.05)

The effect of TMX treatment on the mRNA expression of Bax, Bcl-2, and P53 genes was investigated by qRT-PCR in ovary tissues (Fig. 7). Compared to the untreated group, a significant downregulation was noticed in Bcl-2 levels associated with a non-significant change between two administered doses, whereas Bax expression showed non-significant alterations relative to the untreated group. The Bax/Bcl-2 ratio in TMX-treated rats showed a significant elevation by approximately 52 and 47% for low and high doses, respectively, compared to the untreated group. Moreover, the expression of the P53 gene showed upregulation in rats treated with the lower dose. However, the high dose caused a significant downregulation relative to the control group. These results suggest that TMX treatment induced changes in the transcripts of apoptosis-related genes and initiated apoptosis by the activation of the intrinsic apoptotic pathway.

### Altered transcription of apoptosis-related markers in TMX-treated uterus

**Fig. 8:**
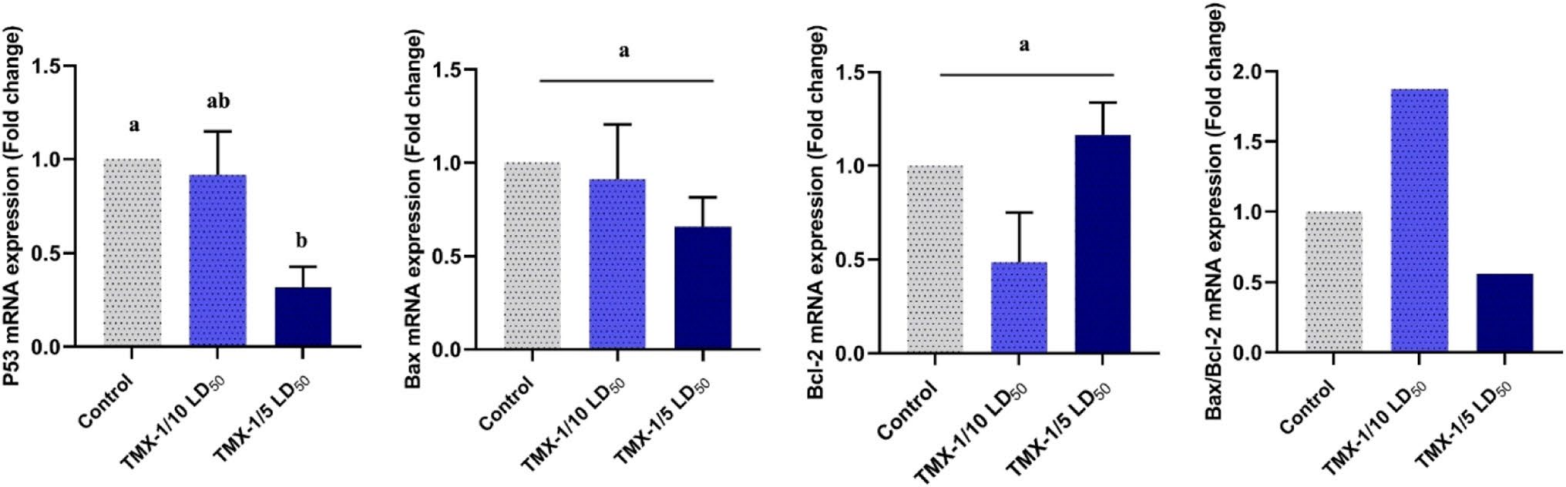
The effect of TMX on the relative gene transcription of Bax, Bcl-2, P53, and the ratio of Bax/Bcl-2 means in uterus tissues. Results were normalized to the GAPDH gene and calculated as a relative fold-change to the untreated group. Data were represented as mean ± SD (*n* = 5) and significance was represented by different letters (*P*˂0.05)

The effect of TMX treatment on the mRNA expression of Bax, Bcl-2, and P53 genes was investigated by qRT-PCR in uterine tissues (Fig. 8). Compared to untreated rats, Bcl-2 levels were significantly (*P*˂0.05) downregulated in the lower dose-treated group; however, the high dose exerted a non-significant upregulation, whereas Bax expression was dose-dependently downregulated with a significant alteration in the high dose treated group relative to untreated one. The Bax/Bcl-2 ratio showed a significant elevation (~55%) in TMX-treated rats (low dose), while the high dose revealed a decrease in ratio by about 45% compared to the untreated group. Moreover, the expression of the P53 gene showed a dose-dependent downregulation with a significant change in the higher dose-treated group relative to untreated one. These results suggest that TMX treatment induced changes in the transcripts of apoptosis-related genes and initiated apoptosis by activating of intrinsic apoptotic pathway in the lower dose-treated rats.

## Discussion

Thiamethoxam is a neonicotinoid insecticide that belongs to the nicotinyl compound subclass. Previous animal research have shown that neonicotinoid insecticides reduce mammalian reproduction despite having a reduced affinity for mammalian nAChRs (Kapoor et al. 2011; Bal et al. 2012). When compared to the control group, the weight gain change in the TMX-treated rats was significantly decreased. This might be due to the insufficient nutrient absorption occured by decreasing feed consumption and the rapid effects of TMX on the gastrointestinal system. These outcomes were matched to previous records (Hassan et al. 2019; Abd-Allah et al. 2022).

In this study, lower dose of TMX caused a significant increase in serum FSH; however, the higher one decreased the FSH levels significantly. This result is consistent with the findings reported by Recio et al. (2005). Furthermore, a non-significant increase was observed in serum ED in animals exposed to both TMX doses. The hypothalamic-pituitary complex and sex organs produce the reproductive hormones (FSH, LH, ED, and PG) that play a precise and coordinated role in the regulation of folliculogenesis, oogenesis, the estrous cycle, sexual behavior, and ovulation (Sorelle et al. 2019). Female reproduction and fertility require hormonal balance or a proper level of sexual hormones. Changes in estrogen or progesterone levels can upset this balance (Bretveld et al. 2006). The hormonal imbalance in LH, PG, and FSH levels may be due to the effect of generated reactive oxygen species (ROS) by neonicotinoids’ exposure which has been reported to exert these changes in rats (Kapoor et al. 2011; EL-Hak et al. 2022). ROS affects a variety of feminine physiological processes, including egg maturation, fertilization, embryo development, and pregnancy. It is also important in the normal cycling of the ovaries, follicular development, and cyclic endometrial changes (Agarwal et al. 2005; Kapoor et al. 2011). Many pesticides are hydrophobic molecules that bind extensively to biological membranes, particularly phospholipid bilayers, and may cause membrane damage by inducing lipid peroxidation (Uzun et al. 2009; Kapoor et al. 2011). However, many pesticides are also known to be associated with enhanced production of ROS (Güney et al. 2007; Guney et al. 2007). Oxidative stress is defined as an imbalance between pro- and antioxidant species that causes molecular and cellular damage (Conti et al. 2016; Tan et al. 2018). Its effect on the organism is determined by the type of oxidant, the location and intensity of its production, the composition and activities of various antioxidants, and the ability of repair systems (Sivoňová et al. 2007). In biological systems, this is associated with an excess of ROS and/or a lack of enzymatic and nonenzymatic antioxidants (El-Garawani et al. 2020a). The increase in ovarian and uterine MDA in the current study could be attributed to an increase in ROS caused by TMX (Kapoor et al. 2011; Nie et al. 2019). These findings support the hypothesis that lipid peroxidation (LPO) is a molecular mechanism involved in TMX-induced ovarian damage. Moreover, the effects of TMX on bovine oocyte formation, with poor cleavage efficiency and delay the growth of morulae and blastocysts, were reported (Nie et al. 2019). Enzymatic antioxidants superoxide dismutase (SOD) and catalase (CAT) are thought to be the first line of defense mechanism to neutralize free radicals and stop their additional production (Ighodaro and Akinloye 2018; Ra et al. 2023). The SOD enzyme catalyzes the disruption of superoxide radicals, resulting in the formation of hydrogen peroxide, which is then detoxified by the enzyme CAT. The decrease in cellular SOD and CAT enzymes as well as the non-enzymatic antioxidant GSH clearly produces oxidative stress (Aly et al. 2009; Grosicka-Maciąg et al. 2011; Kapoor et al. 2011). The decreased activity of SOD, CAT, and GSH in the present study revealed that TMX exerted severe oxidative stress, which affects the body’s inbuilt mechanisms and subsequently causes ovarian and uterine damage (Kapoor et al. 2011). In addition, the decrease of SOD and CAT activity suggests that TMX may extremely deplete the body’s natural antioxidant system due to oxidative stress in testicular cells (Habotta et al. 2021).

In the present study, ovary of TMX-exposed rats showed a reduction in ovarian follicles and disorganized granulosa layer. In addition to degenerative luteal cells with vacuolation in Corpus luteum, this is consistent with previous work and suggests that the decrease in ovary weight was due to extensive fibrosis and atretic follicles (RAO and Kaliwal 2002; Liu et al. 2021). Furthermore, the uterus of TMX-exposed rats showed degeneration of the circular muscle layer with a high rate of apoptotic figures, circular muscle necrosis infiltrated by lymphocytes, and epithelial hyperplasia in the form of finger-like projections. These are supported by the findings in imidacloprid-exposed rats (Lohiya et al. 2018). The histopathological changes in the ovary and uterus were also found to be associated with increased lipid peroxidation and depleted GSH in female rats (Alchalabi et al. 2016; EL-Hak et al. 2022). Collectively, our results of histopathology may be attributed to the free radical generation and oxidative stress, which disturb the cell membrane lipids (EL-Hak et al. 2022). Such cellular disturbances may lead to DNA damage and/or cell cycle arrest and failure. As a consequence of these irreversible events, the cell progresses to apoptosis (Basal et al. 2020). Apoptosis is a cellular process that is highly regulated by various pro-apoptotic and anti-apoptotic proteins, such as members of the inhibitor-of-apoptosis protein family and the Bcl-2 family which functionally inhibits apoptosis by binding directly to BAX effector proteins. The analysis of apoptosis-related pathways focuses particularly on bcl-2, Bax, and caspases (Lian et al. 2019; Li et al. 2020; Zhang et al. 2022; Nur et al. 2022).

One of the most important proteins at the cell cycle checkpoint is the tumor suppressor protein P53, which can be activated by DNA damage, hypoxia, and apoptosis (Hussain and Harris 1998; Sarıgöl Kılıç and Ündeğer Bucurgat 2018). Pesticides have been shown to cause apoptosis in both extrinsic and intrinsic pathways as they primarily increased mitochondrial oxidative stress mediators and activated the cytochrome-C pathway, leading to intrinsic apoptosis (Chi et al. 2014; Jang et al. 2015). TMX’s toxicological and carcinogenic effects are linked to the regulation of inflammatory cytokines and apoptosis-related protein expression (Banerjee et al. 2001; Li et al. 2015). In response to certain apoptotic stimuli, caspase-3 proenzyme is cleaved and activated (Nicholson and Thornberry 1997). Granulosa cells from atretic, but not healthy, follicles express the caspase-3, which suggests the gonadotropin regulation of this enzyme’s expression during the granulosa cells apoptosis (Boone and Tsang 1998). The current study found a significant dose-dependent elevated levels of caspase-3 protein in ovarian and uterine tissues of TMX-treated rats. This outcomes are consistent with the findings of the previous work (Sara et al. 2016; Gasmi et al. 2019). Through the stimulation of apoptosis and endoplasmic reticulum stress, TMX administration in mice affected ovarian homeostasis and lowered oocyte quality (Liu et al. 2021). These results indicate that TMX delays bovine oocyte progression to MI stage, blocks them at the metaphase I stage, triggers disordered chromosomes and spindles at metaphase II stage, and ultimately results in MII oocytes with poor cleavage ability and inhibited development to morulae and blastocysts (Nie et al. 2019). P53 deficiency reduces cellular viability, lifespan, and chromosomal instability (Lowe and Lin 2000). Herein, the upregulation of P53 gene in ovarian tissues was noticed in TMX-treated rats (lower dose). However, the higher one caused a significant downregulation relative to control group. Moreover, the expression of the P53 gene in the uterus showed a dose-dependent downregulation with a significant change in the higher dose treated group relative to the untreated one. These findings suggest that TMX treatment altered the transcription of apoptosis-related genes and may trigger apoptosis by activating the intrinsic apoptotic pathway. The histopathological figures and elevated Bax/Bcl-2 ratio suggest the event of apoptosis (Elmore 2007). However, the higher dose of TMX-treated uterus revealed a decrease in Bax/Bcl-2 ratio, these differences among the treatments suggest the highly toxic effect of this dose on uterus tissues or may affect the transcriptional status due the higher stress on the organ. Furthermore, the internal cellular dysfunction caused by the treatments may lead to the degradation of mRNA molecules (El-Garawani et al. 2020b). Moreover, results of caspase-3 support the evidence of apoptosis which may undergo other pathway in the higher dose treatments rather than the intrinsic one.

## Conclusion

The exposure of TMX to female rats may have a hazardous impact considering their fertility through induction of oxidative stress, disruption of reproductive hormonal levels, histopathological changes, and altered the expression of apoptosis-related genes in the uterus and ovaries. The results of this study may be useful in determining the risks that TMX poses to reproductive health. Further studies on the teratogenicity and the impact on pregnant females are needed, with an emphasis on the underlying mechanisms.

## Data Availability

All data generated or analyzed during this study are included in this article.
